# ‘Beauty Is No Quality in Things Themselves’: Epistemic Motivation Affects Implicit Preferences for Art

**DOI:** 10.1371/journal.pone.0110323

**Published:** 2014-10-31

**Authors:** Antonio Chirumbolo, Ambra Brizi, Stefano Mastandrea, Lucia Mannetti

**Affiliations:** 1 Department of Social and Developmental Psychology, Sapienza University of Rome, Rome, Italy; 2 Department of Cultural and Educational Studies, University of Roma Tre, Rome, Italy; University G. d'Annunzio, Italy

## Abstract

Art preferences are affected by a number of subjective factors. This paper reports two studies which investigated whether need for closure shapes implicit art preferences. It was predicted that higher need for closure would negatively affect implicit preferences for abstract art. In study one, 60 participants were tested for dispositional need for closure and then completed an Implicit Association Test (IAT) task to measure their implicit preference for abstract (vs. figurative) paintings. In study two, 54 participants completed the same IAT task. In this experiment need for closure was both manipulated by cognitive load and tapped as a dispositional trait. Results of the studies converged in showing that after controlling for other important individual factors such as participants'expertise and cognitive ability, need for closure, both as a dispositional trait and as a situationally induced motivational state, was negatively associated with implicit preference for abstract art.

## Introduction

People differ widely with respect to aesthetic taste in diverse fashions and domains. The old adage ‘Beauty is in the eye of the beholder’, or the famous aphorism of the Scottish philosopher Hume “Beauty is no quality in things themselves: it exists merely in the mind which contemplates them; and each mind perceives a different beauty.” p. 155 [1], provide a good illustration of the fact that ‘beauty’ depends not just on intrinsic features of the object, but also on subjective evaluation. Although aesthetic preferences including art preferences are affected by several cultural and historical factors [2], [3], they are ultimately rooted in psychological processes [4], [5] and have specific neuroanatomical correlates [6], [7]. These findings suggest that art preferences are shaped by both bottom-up factors, such as symmetry and complexity [8], [9] and top-down factors, such as viewer characteristics, for example expertise and personality [8]–[10].

In the research reported here we focused on how psychological variables affect art preferences, by investigating the epistemic motivation (specifically the need for closure) influence on art preferences at implicit level. We firstly review the literature on individual differences and art preferences, then we address the concept of epistemic motivation and need for closure and its relationship with art preferences.

### Individual Differences in Art Preferences

To some extent aesthetic judgments and art preferences depend on personality characteristics. Eysenck investigated in various studies [11], [12] people's aesthetic preferences by asking participants to rank groups of different images (e.g. portraits, landscapes), colors, odors, and geometric figures on aesthetic value. Factor analysis of these data yielded a two-factor model of aesthetic value. One factor was a general ‘objective’ aesthetic appreciation dimension (called ‘T’ factor) that was fairly constant for individuals across domains. According to the author, this factor strongly predicted systematic individual differences in aesthetic preferences: “As applied to persons, this factor is the core reality behind what is generally called ‘good taste’” (p. 100) [11]. When this T factor was taken into account, a second bipolar factor emerged (named ‘K’) that seemed to divide individuals on the basis of preference for ‘formal’ or ‘representative’ pictures [11]. This second factor contrasted a colorful, complex, impressionistic and expressionistic art style with a simpler, more symmetrical, and less colorful realistic art style [12]. This factor turned out to be correlated with personality, as modern art style was generally preferred by the extroverted person, whereas “the introvert tended to prefer the old masters” (p. 268) [12]. High K factor scores were also associated with radicalism (vs. conservatism) and with youth [12].

Palmer and Griscom [13] have recently proposed that ‘preference for harmony’ is the fundamental aesthetic individual difference that underlies Eysenck's T factor. Harmony was defined as regularity, simplicity, and parts that go well together. These authors investigated aesthetic preference for stimuli in four different domains (colors, shapes, spatial configurations and music). They found that variability in preference for harmony was captured by one factor, with strong correlations among preferences across all four dimensions. Individuals' preferences were very consistent, i.e. some individuals liked harmonious stimuli whereas others disliked them.

In this perspective, many studies investigated the preference for abstract and modern art over traditional, representational art, and the relationship between aesthetic preference and personality. Most research has focused on two constructs, namely ‘sensation seeking’ [14] and ‘openness to experience’ [15]. Individuals with high sensation seeking scores tend to prefer surreal art [16], and abstract art [10], [17] over traditional or representational art. Likewise, openness has generally been associated with positive evaluations of abstract art and pop art [18], [19]. Personality traits affecting art preferences also appear to influence related behaviors, including which kinds of museum or art gallery are visited. Mastandrea, Bartoli and Bove [20] investigated the personality of visitors at different museums and found that visitors to a museum of modern art were higher in sensation seeking than visitors to a museum of traditional art. Openness to experience, however, did not seem to play a role in choice of art venue. In addition, visitors to the different museum types reported different motivations for visiting: visitors to the museum of modern art reported more emotional reasons (e.g. pleasure, fun, excitement) and visitors to the museum of traditional art listed more cognitive reasons (e.g. interest, enrichment).

### Epistemic Motivation and Need for Closure

Within the approach of lay epistemology [21], [22] and motivated social cognition [23], epistemic motivation is defined as the process of knowledge construction and can be understood as the general inclination to achieve an understanding of an experience [24]. One of the most studied phenomena in this field is the motivation for non-specific closure of the epistemic process, referred to as Need for Closure (NFC) [25], [26]. NFC represents the desire for stable, solid knowledge in order to avoid uncertainty, and the need to have “a firm answer to a question and an aversion toward ambiguity” (p. 264) [27]. The NFC concept suggests a strong interdependence between the cognitive and motivational aspects of the knowledge formation process.

NFC is a stable individual disposition; some individuals display a systematic proclivity to value closure whereas others tend to avoid closure and prefer openness [26]. Individuals with high NFC are more intolerant of ambiguity and prefer structured and predictable environments. Individual differences in NFC may stem from a variety of factors including cultural and societal norms, socialization and social learning processes, for instance social and cultural environments where confidence in one's own opinions and judgments is rewarded or where order and clearness are appreciated may foster NFC [28].

Level of NFC can also depend on the situation. In particular circumstances in which the perceived benefits of closure are more salient and the perceived costs are reduced - such as time pressure, environmental noise, cognitive load, boredom or the dullness of a particular cognitive task - increase the level of NFC in individuals and groups. In other words, when information processing is rendered more difficult and effortful, individuals are *motivated* to close the epistemic process and attain secure knowledge. By contrast, the need to avoid closure may be increased in conditions where the costs of closure and the benefits of a lack of closure are salient (e.g. accountability, fear of invalidity, evaluation apprehension).

Tendencies to urgency and permanency are consequences of NFC. Urgency is defined as the tendency to seize on early cues; permanency reflects the tendency to freeze on existing knowledge to preserve past and future cognition. Urgency and permanency were shown to affect a wide range of individual, interpersonal and group phenomena mediated by information processing [24], [27], [22], [28].

#### NFC and Art Preference

Epistemic motivation may also influence preference for specific kind of art. One would expect higher levels of NFC to be associated with a dislike of more abstract, metaphorical and ambiguous visual stimuli and hence a preference for more representational, concrete artworks. In general, the processing fluency of a stimulus affects its aesthetic appreciation. The ease with which viewers extract meaning from an artwork (i.e. fluency of processing) is linked to aesthetic response: higher fluency is usually associated with a positive aesthetic evaluation [8], [29], [30]. Meaningfulness is also an important predictor of aesthetic response: individuals tend to like artworks they find meaningful [31]. Empirical research showed that abstract art is generally appreciated less than figurative art [32]. Possibly, the reason is that meaning extraction of abstract art items requires greater cognitive effort, prompting the consequent negative aesthetic evaluation [33]. Given that NFC limits the extent of information processing [34], it is plausible to suggest that it may negatively affect liking for abstract art. In fact, processing ambiguous and poorly defined pieces of art appears to conflict with a desire to attain a quick closure of the epistemic process. Appreciation of abstract art requires more effort in information processing that would misfit to the tendency to urgency induced by NFC and the intolerance of ambiguity displayed by high NFC individuals, providing a possible explanation for a negative affective response to abstract artworks.

Chirumbolo and Mannetti [35] conducted a series of studies to test these hypotheses, investigating the impact of NFC on art preferences. In these studies participants previously tested for dispositional NFC were asked to rate abstract and representational paintings on semantic differential anchored by pairs of adjectives measuring *pleasantness* (e.g. pleasant-unpleasant, attractive-unattractive) and *comprehension* (e.g. understandable- incomprehensible). Participants were also asked how much they like each painting. Results converged in showing that even controlling for familiarity, higher NFC scores were negatively correlated with liking and preference for abstract paintings, but not with comprehension. No correlation between NFC and ratings of representational paintings were found. These findings were reliably replicated and extended to other domains such as advertising [36]. Moreover a situational manipulation of NFC (cognitive load) was also shown to affect preferences: in the high NFC condition, participants disliked abstract advertising posters more than conventional advertising posters [36]. In a similar vein, Wiersema, van der Schalk and van Kleef [37] showed that high NFC individuals liked a play with an open ending less than low NFC individuals, (study one), and preferred figurative paintings to abstract paintings (study two). Additionally, in a high NFC condition (under time pressure), participants also liked figurative paintings more than abstract paintings (study three) [37].

### Implicit Art Preferences

De Houwer, Teige-Mocigemba, Spruyt and Moors [38] define an implicit process as the outcome of a measurement procedure which can have various features of automaticity, using the decompositional model of automaticity proposed by Bargh [39]. Different measurement techniques like priming and IAT (Implicit Association Test) show individuals' preferences without the need of asking them directly. According to Fazio and Olson [40] it is more appropriate to consider the measures of attitudes as implicit; it can be the case that individuals are aware of their attitudes, even if the priming or IAT does not measure the awareness specifically.

Although implicit processes are in general the subject of considerable research attention in the field of social cognition [41], they remain almost unexplored in the field of aesthetic appreciation. Most of empirical research into aesthetics has privileged the use of explicit evaluations. The predominance of self-report or explicit measures in empirical aesthetics research has led to a focus on processes characterized by cognitive control, intention and awareness and neglect of those based on automatic or implicit cognition.

In the first published research on implicit art preferences, Mastandrea, Bartoli and Carrus [42], investigated evaluation of different art (figurative art vs. abstract) and architectural (classical architecture vs. contemporary) styles using the Implicit Association Test paradigm (IAT). They found that reaction times were faster in the compatible task (figurative art with positive words and abstract art with negative words) compared to the incompatible task (figurative art with negative words and abstract art with positive words). Participants were faster to categorize positive words in association with figurative art and classical architecture than in association with abstract art and contemporary architecture. These results provide support for the hypothesis that aesthetic evaluations of stimuli in domains such as art and architecture can also be activated implicitly.

Since this study was published, several scholars have used the IAT procedure to explore implicit processes in the context of aesthetics and preferences. Pavlović and Marković [43] wanted to explore the implicit aesthetic evaluation using more specific categories of artistic stimuli (rather than simply categorizing them as ‘figurative’ or ‘abstract’) so they considered a semantically homogenous category - artistic portraits - and a category that lacks semantic information - abstract art. Using the IAT task, they found that people implicitly preferred representational art by Leonardo to works by Dubuffet and abstract art by Klee over abstract art by Kline, in accordance with positive explicit evaluations of Leonardo and Klee paintings obtained in previous studies.

In another study of implicit preferences using the IAT task Makin, Pecchinenda and Bertamini [44] investigated preference for two different types of symmetry, i.e. reflection and translation. They found that polygons with reflection symmetry were preferred to polygons with translation symmetry. In further research, Bertamini, Makin and Rampone [45] showed that aesthetic responses to symmetry involve both positive valence and high arousal, and that these emotional responses arise from the perceptual simplicity of symmetry, which is consistent with the fluency model of aesthetics [30].

In other research, Mastandrea and Maricchiolo [46] extended the study of implicit evaluations to another art-related field, such the design, and explored the role of expertise as a moderator of implicit appraisals. The stimuli were classical and modern design objects, such as chairs. Two groups of participants with different levels of expertise in design (laypeople and experts) performed an IAT task. Findings showed that for laypeople there were no significant differences in latency between compatible (classic chairs/positive words and modern chairs/negative words) and incompatible (classic chairs/negative words and modern chairs/positive words) tasks whereas expert participants were significantly faster on the incompatible task than the compatible task. These results indicated that implicit preferences for classic and modern objects were affected by expertise: experts showed an implicit aesthetic preference for modern objects whereas laypeople did not show a preference for one style over another. This study was one of the first attempts to demonstrate differences between laypeople and experts in aesthetic preferences at an implicit level.

Implicit aesthetic assessment plays an important role in aesthetic experience more generally. Implicit processes are the first phase in several cognitive models of aesthetic appreciation [29], [47]. In the initial stages of the aesthetic exploration people start building their evaluation on a basis that influences their later aesthetic experience. During the initial stages of evaluation, some responses can be triggered implicitly, depending on the mental schemata with which the observer is endowed. Aesthetic appraisal of a work of art might start with uncontrolled and unaware processes, before controlled and conscious processes are activated.

### Aim and Hypotheses

Research on implicit aesthetic preferences is a very recent development and no study has yet investigated the influence of a psychological characteristic or personality trait on implicit art preferences. Building on promising results at the explicit level [35]–[37], two studies investigating the relationship between NFC and art preference were conducted, extending previous findings to the implicit level of analysis. It was hypothesized that the well-known automatic preference for figurative over abstract art is indeed driven by NFC. Therefore, it was predicted a negative association between NFC (both as an individual disposition and as a situational variable) and implicit attitudes to abstract paintings. The design of these studies was also intended to address the shortcomings of previous studies on this topic which failed to rule out alternative explanations for the results [35]–[37].

It is possible that the relationship between NFC and art preferences could be accounted for by other factors such as expertise (e.g. training and education in art) and cognitive ability (e.g. abstract reasoning). As we noted earlier processing fluency tends to generate more positive aesthetic evaluations [29]. Higher processing fluency can be due to several factors, one of which is the difficulty/ease with which a given stimulus is processed; this may be why abstract art attracts lower absolute aesthetic ratings. Other factors influencing processing fluency are the accessibility of conceptual knowledge and cognitive ability, both of which can drive understanding of the stimulus and the aesthetic pleasure of the experience [8]. It can therefore be argued that because abstract artworks require more effortful processing, individuals with more expertise and higher cognitive ability are more likely to have positive aesthetic feelings for them. Background knowledge and superior reasoning ability might both enhance processing fluency, thereby promoting a better understanding of the work and increasing the likelihood of a favorable aesthetic evaluation of abstract artworks.

These studies extend previous findings about the relationship between NFC and aesthetic preferences to the implicit level. They are also the first investigation of the hypothesis that implicit art evaluations can be explained by a psychological variable such as dispositional and situational NFC.

## Study One

In the first study, the relationship between dispositional NFC and implicit art preference was investigated. It was assumed that a higher NFC would be negatively associated with implicit preference for abstract rather than figurative paintings. We also aimed to show that this effect was due to NFC and not to other confounding factors such as art expertise and cognitive ability.

### Method

#### Participants

Participants were 60 women, aged between 19 and 30 years old (M = 29.08; SD = 2.94), from the Faculty of Medicine and Psychology at the Sapienza University of Rome. All participants had normal or corrected to normal vision. None of them had received specific training in art or architecture. They all volunteered to participate in the experiment. The study and the consent procedure were approved by the Ethics Committee of Psychology Research of Sapienza University (n°X-CED01). Participants provided oral informed consent after reading a form. A written consent was not asked as we wanted to guarantee the anonymity of our participants who were also our students.

#### Procedure and Materials

Participants were welcomed to the laboratory and completed a booklet containing measures of NFC scale, cognitive ability and art expertise. Then they completed an IAT in order to tap their implicit attitudes to abstract and figurative paintings. The IAT [48] is a paradigm used to assess the strength of the association between concepts and their affective attributes, using reaction times. This method does not require conscious awareness of the association and has been used in several areas of psychological research, including social cognition. Technically, the IAT is a computerized method used to estimate the strength of the association between a target concept and a valence attribute indirectly via reaction times (faster when association is stronger) on a double categorization task. The IAT uses one target category (e.g. figurative pictures), one contrast category (e.g. abstract pictures), one target attribute (e.g. positive), and one contrast attribute (e.g. negative), each represented by a series of stimuli (e.g. pictures of paintings, words). In our study the pictures and words were presented one at a time in a random order on the computer monitor. Participants had to classify pictures and words (the stimuli) into four categories, by pressing two keys (‘e’ or ‘i’) on the computer keyboard. In this study there were two stimulus categories, *figurative art pictures* and *abstract art pictures* each comprising five exemplars, and two attribute categories, *positive words* and *negative words*, comprising respectively five words with a positive meaning (*beautiful, relaxing, attractive, peaceful*, *interesting*) and five with a negative meaning (*ugly, stressful, repellent, chaotic, boring*). The instruction sheet, which participants read before performing the IAT task, gave a couple of examples of the different art styles (figurative and abstract). In the debriefing phase none of the participants reported having problems identifying the artistic styles. Pictures were selected and pre-tested in a pilot study to ensure that they were clearly identifiable as ‘abstract’ or ‘figurative’ in style and all the images were high quality digital reproductions (see Table 1). The characteristics of the pictures were assessed in a pilot study in which 35 participants evaluated 20 pictures (10 figurative, 10 abstract) presented on a computer monitor without a time limit (see Mastandrea et al., 2011). The pictures were evaluated using a seven-point semantic differential scale for distinguishing between figurative and abstract items. The 10 pictures which obtained the most extreme evaluations on the figurative-abstract dimension were selected for use in this study. The mean score for the selected figurative images was *M* = 1.7 (*SD* = 0.7); the mean score for the selected abstract pictures was *M* = 6.3 (*SD* = 0.8). A *t*-test confirmed that the groups differed significantly on the figurative-abstract dimension, *t* (34)  = 4.04, *p* = .001.

**Table 1:** Selected paintings for the study.

Selected figurative paintings:	Canaletto, *Il campo di Rialto (Venezia),* c. 1758–63
	Daubigny, *Lavandières au bord de l'Oise*, 1874
	Daubigny, *Aldeia de Optevoz*, 1852
	Vermeer, *Street in Delft*, c. 1657–1658
	Constable, *A Cottage in a Cornfield*, 1817
Selected abstract paintings:	Kandinsky, *Composition VIII*, 1923
	Paul Klee, *Highways and Byways*, 1929
	Kasimir Malevich, *Suprematist painting*, 1916
	Jackson Pollock, *Enamel and aluminum paint on canvas (Number 1)*, 1949
	Hoffman, *Indian Summer*, 1959

The experimental task consisted of seven blocks of trials presented in succession: five twenty-trial blocks were used to familiarize participants with the various stimuli (pictures and words); the other two blocks (blocks 4 and 7) were sixty-trial test blocks: the compatible task and the incompatible task. In the compatible test block participants had to pair figurative art images (e.g. pictures of paintings by Canaletto or Constable) with positive words (e.g. ‘beautiful’, ‘relaxing’) with the left key and pair abstract art images (e.g. pictures of paintings by Kandinsky or Klee) with negative words (e.g. ‘ugly’, ‘stressful’) with the right key. In the incompatible test block participants paired abstract art with positive words using the left key and figurative art with negative words using the right key.

It is important to note that the terms ‘compatible’ and ‘incompatible’ are not used in absolute terms, but are relative to the main hypotheses put forward in our study. The compatible task reflects the idea of an easier association between figurative art with positive words and abstract art with negative words on the one hand; whereas, the incompatible task should reflect the idea of a more difficult association (figurative art/negative words and abstract art/positive words), on the other hand.

Participants were asked to respond as quickly and accurately as possible. When a categorization error was made a red ‘X’ appeared in the center of the screen and the participant had repeat the trial; reaction times of errors were not counted, but the correction was important so that the categorization task was made clear. To monitor possible learning effects, half of the participants performed the compatible task (figurative/positive and abstract/negative) in block 4 and the incompatible task (figurative/negative and abstract/positive) in block 7. For the remaining participants the presentation order was reversed. The software *Inquisit 3* (2011) [49] was used to carry out the experiment and record reaction times.

The dependent variable in this study, i.e. implicit art preference, was operationalized as IAT score, which was based on reaction times to each single stimulus, pictures (figurative and abstract) and words (positive and negative). IAT scores were calculated using the D6 algorithm (deletion of latencies below 300 ms and above 10,000 ms, errors replaced with the mean of the correct responses in the corresponding block plus a 600 ms penalty) developed by Greenwald, Nosek and Banaji [50], . The mean of reaction times for this study was 1076.08 ms (*SD* = 424.65). The final score was computed such that higher scores reflected an implicit preference for abstract paintings and lower scores an implicit preference for figurative art. In this study IAT score had an alpha of .90.

Expertise was measured using two items; participants reported the amount of art education they had received and how easily they were able to recognize and identify artworks by famous artists. Respondents answered both questions on a scale from 1 (‘none’ or ‘very difficult’) to 5 (‘a lot’ or ‘very easily’). Score on these items were highly correlated and so were averaged to give a reliable composite index (alpha  = .78).

Cognitive ability was measured by mean of 15 items taken from the reasoning sub-scale of the 16PF-5 Questionnaire [52]. The procedure is typical for a cognitive test: each item consists of a problem with four possible answers, only one of which is correct; one point is given for a correct answer and zero points for an incorrect answer. Higher scores indicate better abstract thinking skills and higher general mental capacity; lower scores indicate lower general mental capacity and reduced ability to handle abstract problems.

### Results and Discussion

Correlations and descriptive statistics for the variables are given in Table 2. Participants tended to exhibit an implicit preference for figurative art over abstract art, replicating the results of previous studies [42]. Implicit preferences for abstract paintings were significantly negatively correlated with NFC, but not cognitive ability or art expertise, whereas cognitive ability was correlated with art expertise and negatively correlated with NFC.

**Table 2:** Study one: Means, standard deviations and correlations among variables.

	1	2	3	4	Mean (SD)
1. Expertise	1				2.55 (.59)
2. Cognitive ability	.25*	1			9.81 (2.20)
3. Need for closure	−.08	−.30**	1		4.09 (.70)
4. Implicit preference	−.07	.13	−.36**	1	−.32 (.47)

Notes. *N* = 60.

*p<.05;

**p<.01.

A hierarchical regression analysis was conducted in order to test the hypothesis that implicit preference for figurative over abstract art was driven by NFC (see Table 3). In the first step, expertise and cognitive ability were included in the equation. Neither variable was a significant predictor, *R* ^2^ = .03, *F*(2, 57)  = .84; n.s. In the second step, NFC was added to the equation, and proved a significant predictor, *R* ^2^ = .14, *F* (3, 56)  = 3.13; *p*<.05 (see Table 3). As expected, NFC was negatively associated with implicit preference for abstract art and this effect was not accounted for by the potential confounding variables investigated, expertise and cognitive ability.

**Table 3:** Study one: Results of the hierarchical regression analysis.

Predictors	Implicit preference
Step1	*Beta*
Expertise	−.10
Cognitive ability	.16
Step2	
Expertise	−.11
Cognitive ability	.05
Need for closure	−.36**

Notes. *N* = 60. Coefficients are standardized beta.

*p<.05;

**p<.01.

## Study Two

The second study was intended to extend the results of study one and focused on NFC as a temporary motivational state induced by specific circumstances. Whilst the impact of dispositions on aesthetic preferences has received considerable research attention, the influence of situational factors on aesthetic preferences has attracted much less attention, although there have been a few exceptions [36], [37], [53]. In particular there is a lack of research on the influence of situational factors on implicit art preferences, which this study was designed to address. In addition to measuring the same variables as in study one, we also manipulated NFC via cognitive load. Situations that render information processing very costly (e.g. time pressure, cognitive load) increase the salience and the benefits of a quick closure of the epistemic process [22], [25]. Therefore, people that have to make judgments or express evaluations in situations of high (vs. low) cognitive load are deemed to be in a high (vs. low) NFC condition [22].

In this study participants were randomly assigned to the high NFC condition (i.e. cognitive load) or low NFC condition (no cognitive load) and then completed the IAT task to measure their implicit art preferences. We hypothesized that after controlling for expertise and cognitive ability, implicit preferences for abstract over figurative art would be negatively related to situational NFC. Dispositional NFC was also measured. It is plausible that the effect of situationally induced NFC on implicit art preference would be moderated by dispositional NFC. However, it is important to emphasize that previous studies of explicit attitudes have tested a similar hypothesis and have consistently found that dispositional and situational NFC have independent effects on explicit aesthetic preferences [36], [37]. That is, the interaction between these two variables always came to be not significant. Would this result be extended also to implicit art preferences? In line with these findings, we will test whether dispositional and situational NFC have an additive or an interactive effect on implicit art preferences.

### Method

#### Participants

Participants were 54 female students, aged between 19 and 28 years old (M = 21.50; SD = 1.82), from the Faculty of Psychology at the Sapienza University of Rome. All participants had normal or corrected to normal vision. None of them had received specific training in art or architecture. They all volunteered to participate in the experiment. The study was approved by the Ethics Committee of Psychology Research of Sapienza University (n°64-CED01). Participants provided oral informed consent after reading a form. We did not ask for written consent as we wanted to guarantee the anonymity of our participants who were also our students. The consent form was presented by one of the experimenters who also checked that all participants had read and understood its content. The ethics committee approved this consent procedure.

#### Procedure and Materials

Expertise, cognitive ability and dispositional NFC were measured the same way as in study 1. Situational NFC was induced via cognitive load, for similar manipulation see [34], [54]. Participants were randomly assigned to either the high cognitive load condition or the low cognitive load condition. Participants in the cognitive load condition (i.e. high NFC) memorized nine numbers, those in the low load condition memorized one number. The numbers to be memorized appeared on a computer screen for 20 seconds and participants were informed that recall would be tested at a later stage. Participants then completed the IAT task which was administered using the same procedure as in study 1. After completing the IAT task participants were asked to write down the number combination they had memorized earlier; only participants who correctly remembered the number combination were included in the experimental group. Mean reaction times for this study were similar in the high and low cognitive load conditions (*M = *902.20 ms, *SD* = 437.25; *M* = 867.70 ms, *SD* = 415.75 respectively; *t* (52)  = 0.30, n.s). Error rates were also similar in the high and low cognitive load conditions (7% and 5% respectively; *z* = 0.39, n.s).

### Results

Correlations and descriptive statistics for the variables are given in Table 4. As in study one participants showed an implicit preference for figurative over abstract paintings. Implicit preference for abstract paintings was positively correlated with expertise; cognitive ability was not correlated with any of the other variables. More importantly, as predicted implicit preference for abstract art was negatively correlated with NFC both as a disposition and a situationally induced state.

**Table 4:** Study two: Means, standard deviations and correlations among variables.

	1	2	3	4	5	Mean (SD)
1. Expertise	1					2.78 (.74)
2. Cognitive ability	.09	1				10.48 (2.07)
3. Need for closure_dispositional	−.09	.10	1			4.20 (.52)
4. Need for closure_situational	−.21	−.02	−.06	1		= = =
5. Implicit preferences	.32*	.06	−.27*	−.59**	1	−.35 (.58)

Notes. *N* = 54

*p<.05;

**p<.01.

A moderation regression analysis was conducted to test the hypothesis (see Table 5), using the procedure described by Aiken and West [55]. Continuous predictors were standardized before computing the interaction term and the regression analysis. In the first step, expertise and cognitive ability were included in the equation. Expertise was found to be a significant predictor of implicit preference for abstract paintings, *R* ^2^ = .10, *F*(2, 51)  = 2.98, *p*<.05. In the second step, dispositional and situational NFC were added to the equation, both were found to be significant predictors of implicit preference, *R* ^2^ = .48, *F*(4, 49)  = 11.31, *p*<.001). As can be seen from Table 5, NFC was negatively associated with implicit preference for abstract paintings both as a situationally induced state and a disposition, and this effect was not accounted for by the potential confounding variables expertise and cognitive ability. The interaction of dispositional and situational NFC was tested in step 3 to detect any moderation effects, and it proved non-significant.

**Table 5:** Study two: Results of the moderated regression analysis.

Predictors	Implicit preferences
Step1	*B*
Expertise	.17*
Cognitive ability	.01
Step2	
Expertise	.09
Cognitive ability	.03
Need for closure_dispositional	−.62**
Need for closure_situational	−.16**
Step3	
Expertise	.09
Cognitive ability	.04
Need for closure_dispositional	−.62**
Need for closure_situational	−.17**
Dispositional NFC × Situational NFC	.02

Notes. *N* = 54; NFC  =  need for closure.

Situational need for closure was coded as 0 =  low, 1 =  high.

Coefficients are unstandardized B.

*p<.05;

**p<.01.

This pattern of findings indicated that high NFC, whether dispositional or situationally induced, was associated with an implicit dislike for figurative paintings relative to abstract paintings. Dispositional and situational NFC exerted independent effects on implicit art preferences and situational NFC had a greater impact than dispositional NFC.

## General Discussion

These studies were designed to demonstrate that implicit relative evaluations of abstract and figurative paintings depend on epistemic motivation. In two experiments we investigated how a specific type of epistemic motivation, NFC, influenced implicit art preferences. It was hypothesized that higher NFC would negatively affect implicit preference for abstract over figurative art. The studies produced convergent results, indicating NFC, both dispositional trait and situationally induced, was negatively associated with implicit preference for abstract over figurative art. Importantly, these findings emerged after controlling for other relevant individual characteristics, namely expertise in art and cognitive ability. This latter finding is particularly important as there is evidence that dispositional NFC is correlated with cognitive ability [56], a result we replicated in study one. Other research on explicit aesthetic preferences failed to take into account this possible confounding factor [36], [37]. In these studies it was shown that implicit art preferences are related to NFC (both dispositional and situational) but not cognitive ability.

It seems self-evident that preferences for different types of art are influenced by multiple factors. Cultural and social factors may well play a role in explaining why particular types of art are considered to have beauty and value [2]. The intrinsic characteristics of the object may also be important [8]. In the end, however, whether a piece of art is appreciated or not depends heavily on a subjective evaluation process. ‘Beauty’ is not an intrinsic characteristic; judgments about beauty are individual and subjective and depend on psychological factors.

Previous research established that variability in art preferences is captured by a bipolar K factor [11]. One pole represents liking for abstract, unstructured, complex, colorful, expressionistic forms of art (e.g. paintings, sculptures) and the opposite pole represents liking for simpler, more conventional, less complex, less colorful, classical figurative forms of art. Convergent results indicate that individuals tend to prefer classical and figurative art to modern and abstract art [32], [57], [58]. Abstract art is commonly judged to be more complex, more difficult, less typical and less familiar than figurative art [42]. Mastandrea and colleagues [42] showed that aesthetic evaluations could be implicit, occurring in the absence of awareness of their content, which opened up a new and promising line of research, use of implicit measures to study aesthetic and art preferences. In line with findings obtained using explicit measures, results revealed that individuals tend to have implicit preferences for figurative rather than abstract art [42].

This preference is often explained in terms of processing fluency. There is a connection between enjoyment and appreciation of an object and the quantity and the quality of features in the object, i.e. its typicality, familiarity and complexity. Aesthetic evaluations are usually more positive if processing of the object is more fluent. It was argued that people tend to prefer figurative art over the abstract art (implicitly as well as explicitly) because abstract art is more difficult to process as it is less prototypical, more complex and less familiar [42]. Prototypicality refers to the degree to which an object is representative of a class of objects. Prototypical objects tend to be processed faster and more easily than non-prototypical objects, and therefore are evaluated more positively. Figurative pieces of art are usually considered more typical than abstract ones and this may explain why they tend to be appreciated more. Complexity in art can related to the number of elements in a work of art and their perceptual organization. People without art training tend to prefer simpler, more symmetrical configurations, whereas people with art training prefer complex and asymmetrical configurations [59]. Figurative and classical art are more symmetrical and less complex than abstract and modern art and therefore tend to be preferred. Finally, familiarity refers to how familiar or novel a given object is. Familiar items are processed faster and therefore tend to be preferred to more novel ones. Usually individuals without art expertise (the large majority of participants in psychological studies) are more familiar with classical and figurative art and therefore tend to appreciate them more than other types of art.

The preference, explicit and implicit, for figurative rather than abstract art is consistent and robust, but can only partially be explained by reference to intrinsic characteristics. Other lines of research have demonstrated the existence of relationships between individual characteristics and preference for particular intrinsic characteristics in art objects. Individuals with a preference for abstract art tend to be of higher socioeconomic status, have a higher level of education and often have more training in visual arts [18]. Relative enjoyment of different types of art is also related to specific personality traits, such as openness [12], [18], [19], sensation seeking [10], [17], and to cognitive-motivational variables such as NFC [35]–[37].

This paper represent the first time that research on the influence of psychological variables on art preferences has been extended to the implicit level. We also considered the effect of situational characteristics on implicit art preferences, an aspect of evaluation that has often been overlooked in empirical research on art [53]. Our results indicated that *implicit* evaluation and hence appreciation of a piece of art (figurative or abstract) strongly depends not only on the characteristics of the object but on the characteristics of the individual and the situation in which he or she makes the evaluation.

Previous research showed that motivation to reduce uncertainty was related to rejection of creative ideas and novel stimuli more generally [60]. However, since a figurative masterpiece could be considered as creative as an abstract masterpiece, our two studies go beyond this finding by showing that implicit rejection is related to the type of stimulus, with abstract stimuli (but not figurative stimuli) that is more likely to attract negative responses. This negative response can be explained by reference to the motivational-cognitive mechanism of the NFC: under conditions which induce high NFC individuals are *motivated* to close rapidly the epistemic processes of knowledge formation intended to make a judgment or evaluation. Because information processing is more costly and effortful, the desire for unambiguous and stable knowledge predominates and anything which runs counter to this is perceived as unpleasant and displeasing. Under these circumstances ambiguity and novelty may make individuals feel uncomfortable. In this sense, processing ambiguous abstract artworks plainly appears to conflict with NFC, because it runs counter to the desire for rapid closure of the epistemic process. If negative aesthetic evaluations of abstract art are assumed to be due to the greater effort required to extract meaning from them, it follows that appreciation of abstract art is incompatible with the urgency tendency induced by situational NFC, and with high NFC individuals’ intolerance of ambiguity. This would explain why abstract art attracts more negative implicit responses from individuals with high dispositional NFC and in situations which induce NFC.

A limitation of this research must be acknowledged. The results of study 2 are open to an alternative explanation. Although previous research consistently showed that cognitive load can increase NFC [34], [54], [61], [62], it also reduces the available working memory capacity of participants, which means that the effect of cognitive load on implicit art preferences can be accounted for by a change in NFC or a change in cognitive processing capacity or both. However, we emphasize that the results of studies which have employed this kind of NFC manipulation (i.e. manipulations of cognitive load to increase NFC) converge with those of experiments using manipulations intended to induce a need to *avoid* closure, such as accountability, fear of invalidity, desire for accuracy [25]. It is also relevant that dispositional NFC also predicted implicit art preferences. All things considered, we may conclude that NFC predicts implicit art preferences regardless of the interpretation of the effects of cognitive load; nevertheless, future research should attempt to rule out this possible alternative explanation, for example by employing a different manipulation of NFC.

Dispositional and situationally induced NFC had an additive effect on implicit art preferences, as they both contributed independently. However situationally induced NFC was a stronger predictor of preference than dispositional NFC. Whether an individual likes or dislikes an abstract painting appears to depend strongly on *temporary* epistemic motivational state, which is influenced by conditions which affect NFC, such as cognitive load, time pressure, low accountability, noise, dullness of the task etc. Regardless of dispositional NFC, when people have an epistemic motivation to reduce uncertainty and arrive rapidly at a stable judgment they tend to reject stimuli that are ambiguous and ‘fuzzy’, as abstract paintings tend to be. Curators of exhibitions of modern and abstract art should take into account environmental factors which may induce greater NFC in visitors and thus negatively affect viewers’ implicit evaluation of the artworks [63]. If ambiguity of an abstract painting can lead to a consequent lack of understanding, museum curators should make more effort to help visitors understand abstract art, using texts, labels, captions and other kinds of explanations to make the work more compatible with viewers’ NFC and prevent them having a frustrating experience.

In conclusion, NFC, both as a trait and as a motivational state, contributes to a relative lack of appreciation for abstract art, at the implicit level. An important issue for future research is whether implicit preferences mediate the relationship between NFC and explicit evaluation of art. This research, in common with the vast majority of studies in the field, was conducted in the laboratory; it would be interesting to explore aesthetic preferences in ecological context, in the places where art is usually encountered, for example museums. Do visitors with high NFC prefer going to museums of traditional ancient art where figurative art is displayed? Are museums of modern art frequented more by low NFC visitors? Conducting more studies in real world art settings such as museums and recruiting participants with a genuine interest in the arts, e.g. museum-goers, might produce important insights into the psychology of the arts, particularly how personality is related to choice of art venue.
